# Modulators of the Personal and Professional Threat Perception of Olympic Athletes in the Actual COVID-19 Crisis

**DOI:** 10.3389/fpsyg.2020.01985

**Published:** 2020-08-05

**Authors:** Vicente Javier Clemente-Suárez, Juan Pedro Fuentes-García, Ricardo de la Vega Marcos, María José Martínez Patiño

**Affiliations:** ^1^Faculty of Sports Sciences, Universidad Europea de Madrid, Madrid, Spain; ^2^Grupo de Investigación en Cultura, Educación y Sociedad, Universidad de la Costa, Barranquilla, Colombia; ^3^Didactic and Behavioral Analysis of Sports Research Group (ADICODE), Faculty of Sport Sciences, University of Extremadura, Cáceres, Spain; ^4^Department of Physical Education, Sport and Human Movement, Autonomous University of Madrid, Madrid, Spain; ^5^Faculty Sciences of Education and Sport, University of Vigo, Vigo, Spain

**Keywords:** Olympic, psychological inflexibility, personality, anxiety, stress

## Abstract

The COVID-19 pandemic is now a major global health issue, affecting world population and high-performance athlete too. The aim of the present research was to analyze the effect of psychological profile, academic schedule, and gender in the perception of personal and professional threat of Olympic and Paralympic athletes facing the 2021 Tokyo Olympiad in the actual COVID-19 crisis. We analyzed in 136 Olympic (26.4 ± 6.2 years) and 39 Paralympic athletes (31.8 ± 9.3 years) academic and sport variables, individual perceptions about COVID-19 crisis, personality, loneliness, psychological inflexibility, and anxiety. Paralympic athletes perceived higher negative impact in their training and performance by the confinement than Olympic athletes (+24.18, *p* < 0.005, *r* = 0.60). Neuroticism and psychological inflexibility presented the greatest negative feelings for female athletes (+32.59, *p* < 0.000, *r* = 0.13) and the perception that quarantine would negatively affect their sports performance. Finally professional athletes showed lower values in personality tests (*Agreeableness* factor) about COVID-19 crisis than non-professionals (−40.62, *p* < 0.012, *r* = 0.88).

## Introduction

The COVID-19 pandemic is now a major global health issue, representing the most serious respiratory virus since the 1918 H1N1 influenza pandemic (Casadevall and Pirofski, 2020). Spain is the second most affected nation in the world, after the United States, with 152,446 confirmed cases, and the first in the world in the number of deaths, with 15,238, being the country, with a number of 350, with more deaths of Covid-19 per million people on April 11, 2020 (World Health Organization, 2020). On March 14, the Spanish Government declared a nationwide lockdown ordering people to stay at home (Gobierno de España, 2020).

The COVID-19 crisis is a new threat that put the adaptive mechanisms of the human being, both physiologically and psychologically, through its paces. The human being is a highly adaptative organism, for this reason we developed in our evolution different physiological and psychological defense mechanism that allow us to overcome different eliciting contextual scenarios, but some of these phylogenetic defense system could produce non adaptative behavior in current society and specially in exceptional situations (Clemente-Suárez et al., 2020). In the current global home confinement situation due to the COVID-19 pandemic, most individuals are exposed to an unprecedented stressful situation of unknown duration, being a focus of stress, anxiety, fear, and depression or disrupt sleep due to a negative appraisal of the situation and self-protection behaviors (Altena et al., 2020; Li et al., 2020). The lack of vaccines and the actual no population immunity due to the herd effect increase the feeling of danger and uncertainty in this health crisis (Clemente-Suárez, 2020). The perception of uncertainty and lack of control is a source of psychophysiological variations as sympathetic autonomic branch activity, cortisol, and catecholamine release may increase, allowing different pathologies and psychopathologies, such as anxiety or even depression (Clemente-Suárez et al., 2020). Previous researches found how confinement produce altered immune functions paralleled by changes in stress hormone levels, being dependent to the specific characteristics of the confined environment (Strewe et al., 2015). At psychological level evaluated depression, moods state fluctuations and perception of fatigue were also found (Basner et al., 2014). A recent review highlights the importance of providing effective a rapid information for people in quarantine (in addition to medical supplies) (Brooks et al., 2020). They also suggested that the confinement period should be short, and the duration should not be changed unless in extreme circumstances (Brooks et al., 2020). In Spain, in response to COVID-19 pandemic, confinement has extended more than 6 weeks, and subject to changes.

One of the populations that could suffer more the confinement are athletes, especially professional ones. Their daily routines in where primary outdoor activities counteract with the current situation of home confinement. This fact could be a source of loss of performance adaptions, limiting the acquisition of new abilities as well as limiting their performance improvements. Within this group, the Olympic and Paralympic athletes suffer an even more eliciting situation, since they have been preparing the Tokyo Olympiad for 4 years and their postponement is a more added stressor. This setback and the threat of the Coronavirus can cause the athlete to lose concentration, motivation and the desire to continue preparing for the Olympiad with the same energy as he did up until then. the perception of threat of this virus, the psychological profile of the athlete, as well as their psychological skills (Belinchon-deMiguel and Clemente-Suárez, 2018; Belinchon-Demiguel and Clemente-Suarez, 2018; Belinchón-deMiguel et al., 2019; Sánchez-Conde et al., 2019) would be fundamental to understand their stress response and to define professional interventions on these athletes so that they continue to prepare for the Olympiad at the highest level. Other factors as social support, academic level, gender or cultural differences could also modulate the behaviors in eliciting situations (Bellido et al., 2018; Beltrán-Velasco et al., 2019, 2020) making the knowledge of theses parameters and their relationships important factors in actual moments. Then, in the specific population of Olympic and Paralympic athletes the understand of factors that could be controlled, such as their coping styles and psychological flexibility, their social environment and institutional support, may be the basis to apply specific measures in this exceptional state that we are currently experiencing, allowing therefore, that their sports performance does not suffer negative effects.

Then, the aim of this research was to analyze the effect of academic schedule, gender, the individual perceptions about COVID-19 crisis, and psychological profile (personality, loneliness perception, psychological inflexibility, and anxiety), in the perception of personal and professional threat of Olympic and Paralympic athletes facing the 2021 Tokyo Olympiad in the actual COVID-19 crisis.

## Methods

This study was performed online using the Spanish Google Forms internet platform^1^. A correlational design with incidental sampling for convenience was used. During lockout, this methodology was the most appropriate for obtaining information.

### Participants

In the present study were analyzed 175 Olympic and Paralympic athletes classified or in process to the Olympic Games in Tokyo 2021 (*M* = 27.62 years; SD = 7.33). 136 Olympic (*M* = 26.4 years; SD = 6.2) (Artistic gymnastics: 13, Athletics: 64, Golf: 8, Hockey: 42, Judo: 2, Swimming: 1, Triathlon: 2, Volleyball: 1, Wrestling: 3) and 39 Paralympic athletes (*M* = 31.8 years; SD = 9.3) (Athletics: 8, Basketball: 6, Canoe: 1 Cycling: 4, Football: 1, Judo: 1, Swimming: 9, Table tennis 5, Taekwondo: 1, Triathlon: 2, Weightlifting: 1), gender (72 men, 103 women). Before participation, experimental procedures were explained to all the participants who gave their voluntary written informed consent in accordance with the Declaration of Helsinki. All the procedures were approved by the Commission of Bioethics and Biosecurity of the University of Extremadura (Spain) (approval number: 57/2020).

### Procedure

Due to the COVID-19 crisis and the limitation of free movement, an intentional opinion-type sampling type was used. The call to participate in the study was made through a link sent by the federations of the different sports (De La Vega et al., 2020). The questionnaire was sent to 338 athletes: 230 Olympic athletes, answering 136 (59%), and 108 Paralympic athletes, answering 39 (36%). The participation criteria for the athletes was to be over 18 years of age and to be confined in Spain at the time of the study. Athletes who completed the online survey gave their passive consent to participate at the time of participating in the research. This study was completely voluntary, and no personal data was requested through which the participants could be identified.

Data collection lasted for 15 days (from March 24 to April 12, 2020). At that time all the Olympic and Paralympic athletes were in a situation of confinement by order of the Spanish government.

Firstly, they filled the informed consent and then completed the following items.

Academic and sport related information:

• Academic training (High school, University, Professional).
• At this moment, I am qualified for the Tokyo 2021 Olympics (Yes, No).

Individual perceptions about COVID-19 crisis in a Liker 1–5 scale (De La Vega et al., 2020):

• Level of personal concern about COVID-19.
• Perception of social alarm by COVID-19.
• Control perception level to avoid getting infected by COVID-19.
• Level of personal care to avoid contagion by COVID-19.
• Personal consideration about the postposition of Olympics Games to 2021 by COVID-19?
• I consider that high-performance athletes must comply with the confinement regulations to avoid contagion by COVID-19, even at the risk of diminishing their performance in the short and medium term.
• I consider that, because of the current confinement rules, my options to get my best performance in the next Olympics, in Tokyo 2021 (in the case of qualifying), may be affected.
• I am satisfied with the level of support that, in this context of COVID-19, public institutions have had with athletes with options to qualify for the next Olympics.
• Consider that the current situation generated by COVID-19 has greatly affected my training routines.
Psychological profile, by the following questionnaires:
• Personality by a Brief Version of the Big Five Personality Inventory, where extraversion, agreeableness, conscientiousness, neuroticism, and openness to experiences factors were analyzed on a Likert scale from 1 = “I am completely disagree” to = “I am completely agree” (Rammstedt and John, 2007).
• Loneliness by the short version of the UCLA Loneliness Scale. This scale has three items and it measures the perceived social isolation. It is answered on a Likert scale from 1 = “never” to 3 = “always” (Hughes et al., 2004).
• Psychological inflexibility was measured by the Acceptance and Action Questionnaire-II. It is a 7-item questionnaire where participant must respond in a 1 to 7 scale. This scale allow to analyze the psychological inflexibility considered a transdiagnostic process across psychological disorders (Ruiz et al., 2013).
• Anxiety by the State-Trait Anxiety Inventory (STAI) short form composed by six items responded in a 1 to 4 scale (Marteau and Bekker, 1992).

### Statistical Analysis

The recruited data (N = 175), were grouped on the bases of age (*M* = 27.62; SD = 7.33), gender [N_male_ = 72 (41.1%), N_Female_ = 103 (58.9%)], level of education [N_high school_ = 39 (22.2%), N_Professional_ = 25 (14.3%), N_Universitary_ = 111 (63.5%)], Olympic and Paralympic participation [N_Olympic_ = 136 (77.7%), N_Paralympic_ = 39 (22.3%)], and sport specialty [N_Individual_ = 124 (70.9%), N_Collective_ = 51 (29.1%)]. The inclusion criteria used for the present research was that athletes were actually classified or in process to classify to the Tokyo 2021 Olympic Games.

Before the data analyses, the normal distribution of the dependent variables was tested with the Kolmogorov–Smirnov and Shapiro–Wilk tests. All test results were statistically significant (*p* < 0.005 for all tests). Consequently, nonparametric tests were used. Group differences in the dependent variables were tested with the nonparametric Mann–Whitney U test, which is recommended for smaller samples and might be less sensitive to sample size differences between the groups (Fagerland, 2012). In case of three groups (i.e., age-groups), the Kruskal–Wallis H test, also known as the analysis of variance on ranks, was used. A bivariate correlation analysis between all variables was performed using Spearman correlation analysis *r*, which is used to measure the effect size of differences between variables according to the following thresholds: small (|*r|* = 0.1), medium (|*r|* = 0.3), large (|*r|* = 0.5), and very large (|*r|* = 0.7) (Fritz et al., 2012).

## Results

Descriptive data of all variables are presented in Table 1. The findings suggesting that the neuroticism was greater in female compared to male group (+32.59 points), which was also justified by the U Mann–Whitney test [*Z* = 18.110, *p* < 0.000, effect size (*r*) = 0.13, small]. This trend with higher scores in the group of women is also observed in *Inflexibility*, [+15.34 points, *Z* = 3.900, *p* < 0.048, effect size (*r*) = 0.29, small).

**TABLE 1:** Age, individual perception about COVID-19 crisis and psychological variables.

	**N**	***M***	**SD**
Age	175	27.62	7.00
Personal concern	175	3.67	0.97
Perception of social alarm	175	4.18	0.79
Perception of control to avoid contagion	175	4.15	0.85
Personal care to avoid contagion	175	4.51	0.66
Agreement for the delay of the Olympics	175	4.69	0.71
Agreement with the confinement of high performance athletes	175	4.11	1.05
Altered options for maximum performance	175	2.82	1.28
Satisfaction with institutional support	175	3.00	1.26
Alteration of training routines by COVID-19	175	4.59	0.80
Extraversion	175	5.46	1.67
Agreeableness	175	5.71	1.43
Conscientiousness	175	8.1	1.61
Neuroticism	175	5.12	1.95
Openness to experience	175	7.39	1.82
Loneliness	175	4.17	1.64
Psychological inflexibility	175	14.98	6.27
Anxiety	175	13.15	3.93

Regarding the academic training received, the U Mann–Whitney test reveal that athletes with university studies obtain lower scores than the other groups regarding the assessment that high-performance athletes remain confined [−27.59 points, *Z* = 11.247, *p* < 0.004, effect size (*r*) = 0.85, very large]. This trend with lower scores in the group of university studies was also observed in the perceived support of public institutions [−20.42 points, *Z* = 7.018, *p* < 0.030, effect size (*r*) = 0.53, large]. On the other hand, in the personality test results, differences appear in the athletes of professional training with respect to the other categories. These differences are in the sense of obtaining lower scores on the *Agreeableness* factor [−40.62 points, *Z* = 8.836, *p* <0.012, effect size (*r*) = 0.88, very large]. In *psychological Inflexibility*, the results show higher scores of the group with Secondary Education, compared to the others [+28.62 points, *Z* = 7.028, *p* < 0.030, effect size (*r*) = 0.53, large]. Regarding the type of sport practiced, only significant differences in *Agreeableness* appear. In this sense, individual athletes obtain higher scores than those who practice collective sports [+17.85 points, *Z* = 4.746, *p* < 0.029, effect size (*r*) = 0.35, medium].

When the results are compared between Olympic and Paralympians athletes, three variables appear with statistically significant differences. First, Paralympic athletes have higher scores on the importance they place on the confinement of high-level athletes [+24.18 points, *Z* = 8.017, *p* < 0.005, effect size (*r*) = 0.60, large]. Paralympic athletes also receive more perceived support from public institutions than the Olympic athlete group [+13.56 points, *Z* = 28.457, *p* < 0.000, effect size (*r*) = 0.21, small]. On the other hand, the only differences that appear in the personality variables are those that refer to Paralympic athletes obtain higher *Conscientiousness* scores [+23.73 points, *Z* = 6.944, *p* < 0.008, effect size (*r*) = 0.52, large]. Finally, a correlational analysis is shown in Table 2.

**TABLE 2:** Correlational analysis of study variables.

	**1**	**2**	**3**	**4**	**5**	**6**	**7**	**8**	**9**	**10**	**11**	**12**	**13**	**14**	**15**	**16**	**17**
1. Concern	1																
2. Alarm	0.17*	1															
3. Control	0.08	0.03	1														
4. Personal care	0.21**	0.13	0.51**	1													
5. JJOO delay	0.17*	0.01	0.12	0.16*	1												
6. Sports confinement	0.17*	0.03	0.04	0.13	0.15*	1											
7. Influence performance	0.02	0.05	0.05	–0.07	0.12	−0.28**	1										
8. Institutions support	0.09	–0.03	0.14	0.03	0.15*	0.41**	−0.16*	1									
9. Training routines	0.03	0.09	–0.02	0.07	0.03	–0.08	0.28**	–0.08	1								
10. Extraversion	0.00	–0.02	–0.14	−0.15*	–0.08	–0.05	–0.08	0.05	−0.16*	1							
11. Agreeableness	0.01	0.03	–0.02	0.11	–0.07	0.09	0.17*	0.02	0.01	−0.17*	1						
12. Conscientiousness	0.19*	0.13	0.00	0.18*	0.08	0.03	0.06	0.15*	–0.04	0.01	–0.03	1					
13. Neuroticism	0.11	0.01	–0.10	–0.11	–0.01	0.00	0.28**	−0.16*	0.18*	0.00	0.09	–0.03	1				
14. Openness	–0.10	0.05	0.22**	0.23**	–0.01	0.04	0.04	0.07	0.09	−0.57**	0.03	0.10	–0.03	1			
15. Loneliness	–0.07	–0.02	–0.05	–0.08	–0.02	–0.05	0.172*	0.06	0.20**	−0.20**	0.08	–0.13	0.26**	0.11	1		
16. Psychological inflexibility	0.06	0.01	–0.03	–0.12	–0.10	0.01	0.25**	–0.14	0.21**	–0.08	0.08	−0.23**	0.56**	–0.08	0.42**	1	
17. Anxiety	0.03	0.11	0.05	0.03	–0.07	–0.04	–0.04	–0.04	0.10	−0.21**	0.03	–0.09	0.04	0.12	–0.01	0.02	1
*M*	3.67	4.18	4.15	4.51	4.69	4.11	2.82	3	4.59	5.46	5.71	8.1	5.12	7.39	4.17	14.98	13.15
SD	0.97	0.80	0.85	0.66	0.71	1.06	1.28	1.26	0.80	1.68	1.43	1.61	1.95	1.82	1.65	6.27	3.939
N	175	175	175	175	175	175	175	175	175	175	175	175	175	175	175	175	175

*p ≤ 0.05; **p ≤ 0.01.

## Discussion

The aim of the present research was to analyze the effect of academic schedule, gender, the individual perceptions about COVID-19 crisis, and psychological profile (personality, loneliness perception, psychological inflexibility, and anxiety), in the perception of personal and professional threat of Olympic and Paralympic athletes facing the 2021 Tokyo Olympiad in the actual COVID-19 crisis. We found differences in psychological and social parameters between the athletes analyzed.

The present situation of confinement could be a source of stress that could be perceived and somatised increasing levels of anxiety, resulting from the perception of lack of control to adapt to the contextual demands, the imposition of liberty restriction and the non-voluntary self-isolation perception (Halabchi et al., 2020). Its health impact may be related to the duration of the quarantine (longer periods are associated with poorer mental health, avoidance behaviors, and anger), the fear of infection, frustration, and boredom, inadequate supplies (e.g., water, clothes, accommodation) or inadequate information (Brooks et al., 2020). In the present study we found how anxiety levels were in line with non-pathological population, showing no impact of the quarantine in the anxiety response of Olympic and Paralympic athletes. This fact could be related with the higher cognitive resources and large experience of these high-performance athletes in coping with anxiety contexts as competitions (Belinchón-deMiguel et al., 2019). These characteristics could be also explaining the high punctuation in conscientiousness personality factor, fact related with the aim of to control every aspect of training, nutrition, life… that can finally affect competition. In this line, athletes presented a high perception of social alarm, but the concern about COVID-19 pandemic is medium, probably due to the high control perception to avoid contagion and personal care to avoid contagion.

The Olympic games are the most important sport event for athletes, and much of then depend on this event for their sports scholarships and sponsorships. Therefore, the importance of an Olympiad is extremely high for this population. Even so, there was a high agreement on the suspension of the Tokyo Olympics and the confinement of high-performance athletes. Probably due to the perception that confinement may not adversely affect their options for achieving highest athletic performance, even though alteration of training routines by COVID was high. Nevertheless, athletes with higher studies (university) presented lower agreement with the confinement of high-performance athletes. This fact could be related with a lower understanding of the situation and the no possibility to use the facilities of the high-performance centers that they used to train. Probably university athletes could blame public institutions for the lack of access to sports facilities, perceiving that fact as a lack of support, showing lower support perception from public institutions than athletes with less education.

Olympic and Paralympic athletes require a large investment of physical and psychological effort. The economic and professional future of many of these athletes depends on the result of a single competition that is held every 4 years. This is a major factor of uncertainty, pressure and stress (Kellmann and Günther, 2000). Therefore, having an adaptive and resilient personality, as well as, capable to control anxiety is a key factor in achieving success (Fletcher and Sarkar, 2012). Currently, in addition to all these factors, the COVID-19 pandemic appears as a new factor of uncertainty and lack of control, becoming a new stressor for Olympic and Paralympic athletes. Olympic and Paralympic athletes presented low psychological inflexibility, loneliness, and anxiety values, characteristic psychological profile of these population (Bond et al., 2011; Belinchón-deMiguel et al., 2019). Nerveless, athletes with lower studies (high school) presented higher psychological inflexibility than athletes with higher studies. Avoidance and psychological inflexibility are basic psychological characteristics to be able to overcome any negative event that may present, allowing us to become more adaptively to the context. In this line, previous authors have already found a negative relationship between educational level and psychological inflexibility (Wicksell et al., 2010) a factor that would further strengthen educational programs in professional athletes. Paralympic athletes presented higher scores on the importance they place on the confinement of high-level athletes, provably due to their limitation to train at home, perceiving how confinement could have a greater effect on their performance. Furthermore, and since them receive more support from public institutions than the Olympic, and this support depends on their results, its logical these higher values in this parameter.

Correlation analysis shown how neuroticism personalities perceived that confinement would have a higher impact in their performance, negatively affecting their training routines and feeling low institutional support. The negative emotions associated to this personality trait may be behind this poor adaptive thinking in the confinement situation (Harkness et al., 1995). By contrary, openness trait presented higher control and personnel care perceptions, more adaptative to the confinement than the neuroticism ones. In this line the psychological inflexibility also showed to be related with poor adaptive response to the confinement situation since higher psychological inflexibility presented higher negative perception in their sport performance, training routines and more loneliness feeling, and neuroticism trait. Psychological inflexibility is a psychological factor that has been related to worse states of health and less contextual adaptability (Woodruff et al., 2014). In the case of the present study, we found how high psychological inflexibility athletes experienced the greatest negative feelings, thinking that quarantine may affect them the most. This fact shows us the importance of psychological training in high performance athletes in order to maintain a positive mood and more flexible and positivist coping strategies, fundamental parameters to achieve high sports performance (Mummery et al., 2004).

### Study Limitation and Future Research Lines

One of the limitations of the study was the no control of stress related hormones, fact that could improve the effect of the health crisis in the stress response of athletes. Future research must seek these issues. In this line, as a future research line, it would be interesting to analyze the impact of COVID-19 crisis in different performance level athletes as well as in young categories.

### Practical Applications

The present research showed the impact of the unexpected health crisis due to the COVID-19 and how the government measures as confinement directly impact Olympic athletes. According to the results obtained we could recommend specific program to support Olympic and Paralympic athletes in this difficult and eliciting moment, specially at psychological level. In this line, interventions to improve psychological flexibility and coping would be the most recommended in order to reach an optimal and adaptative psychological status of athletes, allowing them to adapt their personal and professional life to the changing events that are occurring.

## Conclusion

Olympic and Paralympic athletes showed negative perception of confinement with regards to their workouts, but not to their performance, in light of the Tokyo Olympics suspension. The confinement was perceived by Paralympic athletes more negatively than Olympic athletes in their training and performance. Regarding differences between sexes, female athletes perceived negatively in neuroticism and psychological inflexibility, as well as their perception of sports performance incidence. Finally, the personality tests scored lower in professional athletes than in non-professional athletes.

## Data Availability Statement

The raw data supporting the conclusions of this article will be made available by the authors, without undue reservation.

## Ethics Statement

All the procedures were approved by the Commission of Bioethics and Biosecurity of the University of Extremadura (Spain) (approval number: 57/2020). The patients/participants provided their written informed consent to participate in this study.

## Author Contributions

JF-G and MM conceived the study and funding acquisition. VC-S supervised the manuscript. VC-S and RV designed the questionnaire. MM collected the data. JF-G and RV analyzed the data. VC-S, JF-G, RV, and MM interpreted the results of the research and, provided critical revisions and the formal analysis on the successive drafts. RV and JF-G designed the figures and tables. VC-S and JF-G wrote and edited the manuscript. All authors approved the manuscript in its final form.

## Conflict of Interest

The authors declare that the research was conducted in the absence of any commercial or financial relationships that could be construed as a potential conflict of interest.

## Footnotes

1 https://www.google.es/intl/es/forms/about
